# Design and Performance Analysis of Sub-THz/THz Mini-Cluster Architectures for Dense Urban 5G/6G Networks

**DOI:** 10.3390/s25216717

**Published:** 2025-11-03

**Authors:** Valdemar Farré, José Vega-Sánchez, Victor Garzón, Nathaly Orozco Garzón, Henry Carvajal Mora, Edgar Eduardo Benitez Olivo

**Affiliations:** 1 Corporación Nacional de Telecomunicaciones CNT E.P., Quito 170525, Ecuador; valdemar.farre@cnt.gob.ec; 2 Colegio de Ciencias e Ingenierías “El Politécnico”, Universidad San Francisco de Quito (USFQ), Diego de Robles s/n, Quito 170157, Ecuador; dvega@usfq.edu.ec (J.V.-S.); hcarvajal@usfq.edu.ec (H.C.M.); 3 ETEL Research Group, Faculty of Engineering and Applied Sciences, Networking and Telecommunications Engineering, Universidad de Las Américas (UDLA), Quito 170503, Ecuador; nathaly.orozco@udla.edu.ec; 4 Department of Communications, School of Electrical and Computer Engineering, University of Campinas (UNICAMP), Campinas 13083-852, Brazil; ebenitez@unicamp.br

**Keywords:** 5G NR, 6G, B5G, critical use cases, dense urban environment, hotspot, mini-cluster architecture, sub-THz, THz

## Abstract

The transition from Fifth Generation (5G) New Radio (NR) systems to Beyond 5G (B5G) and Sixth Generation (6G) networks requires innovative architectures capable of supporting ultra-high data rates, sub-millisecond latency, and massive connection densities in dense urban environments. This paper proposes a comprehensive design methodology for a mini-cluster architecture operating in sub-THz (0.1–0.3 THz) and THz (0.3–3 THz) frequency bands. The proposed framework aims to enhance existing 5G infrastructure while enabling B5G/6G capabilities, with a particular focus on hotspot coverage and mission-critical applications in dense urban environments. The architecture integrates mini Base Stations (mBS), Distributed Edge Computing Units (DECUs), and Intelligent Reflecting Surfaces (IRS) for coverage enhancement and blockage mitigation. Detailed link budget analysis, coverage and capacity planning, and propagation modeling tailored to complex urban morphologies are performed for representative case study cities, Quito and Guayaquil (Ecuador). Simulation results demonstrate up to 100 Gbps peak data rates, sub 100 μs latency, and tenfold energy efficiency gains over conventional 5G deployments. Additionally, the proposed framework highlights the growing importance of THz communications in the 5G evolution towards B5G and 6G systems, where ultra-dense, low-latency, and energy-efficient mini-cluster deployments play a key role in enabling next-generation connectivity for critical and immersive services. Beyond the studied cities, the proposed framework can be generalized to other metropolitan areas facing similar propagation and capacity challenges, providing a scalable pathway for early-stage sub-THz/THz deployments in B5G/6G networks.

## 1. Introduction

In recent years, global demand for mobile-data traffic has grown markedly, together with the need for higher throughput and ultra-low latency to support a wide range of applications and to connect thousands of devices simultaneously. To meet these requirements, the fifth generation (5G) of cellular systems—5G New Radio (NR)—provides capabilities tailored to specific scenarios such as ultra-high mobility, massive connectivity, and very high traffic loads, outperforming legacy systems like Long Term Evolution (LTE) [1]. Radio network planning (RNP) remains a crucial stage and is considered the primary step in deploying any wireless system, including 5G networks. The services enabled by 5G NR are commonly categorized into three use case services: enhanced Mobile Broadband (eMBB), Ultra-Reliable and Low-Latency Communications (URLLC), and massive Machine-Type Communications (mMTC), all of which are applicable to Internet of Things (IoT) applications.

Beyond 5G (B5G) and sixth generation (6G) networks are expected to deliver data rates up to two orders of magnitude higher than 5G, reduce latency to sub-millisecond levels, and improve energy efficiency by roughly an order of magnitude [2]. Compared to 5G, 6G is expected to deliver substantial gains across several metrics: network capacity may increase up to 100× (one hundred times), while throughput, connection density, and energy efficiency could each improve by up to 10× (ten times). Achieving these advances requires exploring new frequency ranges, notably the sub-terahertz (sub-THz) and terahertz (THz) bands. In this study, the sub-THz band refers to frequencies between 0.1 and 0.3 THz, which bridge the gap between millimeter-wave and true THz communications, whereas the THz band denotes frequencies between 0.3 and 3 THz, typically used for ultra-high-capacity backhaul and short-range links [3]. Projections even consider operation at frequencies up to 10 THz. Within the research community, frequencies exceeding 100 GHz are typically encompassed under the umbrella of THz communications. Access to these bands offers unprecedented spectral resources to support the extreme data rates demanded by emerging services such as extended reality (XR), holographic communications, and the tactile internet [4].

Dense urban settings pose challenges for wireless deployments. Cities such as Quito and Guayaquil face complex propagation conditions driven by geographic constraints, high building density, and specific demographic patterns [5]. The mini-cluster concept has emerged as a promising approach to mitigate these issues by supplying localized, high-capacity coverage that can be integrated with existing 5G infrastructure while laying groundwork for B5G/6G systems. In this context, a mini-cluster is defined as a localized deployment unit composed of multiple small base stations (mBSs), intelligent reflecting surfaces (IRSs), and distributed edge computing units (DECUs), interconnected through short-range THz wireless backhaul links. Each mini-cluster operates as a self-contained subsystem enabling coordinated coverage, beamforming, and computation offloading within a dense-urban 6G network [6,7,8].

In recent years, THz communications have garnered increasing attention as a key enabler for B5G and 6G networks due to their ability to unlock ultra-large bandwidths and support extremely high data rates. With tens to hundreds of GHz of contiguous spectrum available, THz frequencies are uniquely positioned to address the insatiable demand for throughput in dense urban scenarios. Moreover, their short wavelengths permit ultra-dense antenna integration and highly directive beamforming, which helps mitigate interference and enhances spatial reuse. Recent surveys and experimental works have advanced understanding of the practical challenges of THz systems, including circuit and device limitations, molecular absorption, and beam alignment constraints [9]. In parallel, emerging research on IRS-assisted THz networks has shown how intelligent reflections and user–IRS associations can overcome severe path loss and imperfect channel state information (CSI) to improve spectral efficiency [10]. These developments underscore that THz communications are not only a theoretical vision but are gradually maturing to become an integral component of future B5G/6G system designs.

On the other hand, research has further explored the convergence of edge computing and THz communications, emphasizing distributed intelligence at the network edge to mitigate latency and processing bottlenecks. Works such as [11,12] demonstrate how hybrid analog–digital beamforming combined with edge-assisted coordination can enhance throughput and energy efficiency in dense THz networks. Likewise, approaches in [8,10] present multi-IRS and intelligent surface frameworks that complement our proposed mini-cluster approach by enabling dynamic environment-aware reconfiguration. These studies collectively validate the relevance of integrating IRS and edge intelligence for scalable B5G/6G architectures, a strategy that our work extends to realistic urban THz deployment scenarios.

This work proposes a comprehensive design methodology for mini-clusters operating in sub-THz and THz bands, specifically aimed at hotspot and critical-mission use cases in the dense urban environments of Quito and Guayaquil. The objectives are as follows: (1) to establish a link-budget formulation for sub-THz and THz frequencies, (2) to develop coverage and capacity planning strategies for mini-cluster deployment, (3) to analyze propagation characteristics in dense urban scenarios, and (4) to validate the proposed designs through extensive simulations. The primary contribution of this paper lies in the holistic integration of these modeling and simulation elements into a unified framework that bridges the gap between theoretical sub-THz/THz communication models and their practical deployment in real urban environments. Unlike previous studies that address these aspects separately, our methodology simultaneously accounts for link budget, propagation, and capacity interactions—providing a validated, scalable design blueprint for future B5G/6G mini-cluster networks.

While the individual propagation and system models adopted in this work are based on the existing state-of-the-art, the primary scientific contribution of this paper lies in the holistic integration and adaptation of these models into a comprehensive design and validation framework for sub-THz mini-clusters. Our work addresses the unique challenges posed by dense urban environments with complex morphologies, such as those found in Quito and Guayaquil. By synthesizing link-budget analysis, advanced propagation modeling, and system-level simulation, this study provides a validated, scalable methodology that bridges the gap between theoretical models and practical deployment considerations for B5G/6G networks. This integrated approach offers insights that are broadly applicable to metropolitan areas facing similar deployment barriers.

The remainder of the paper is organized as follows: Section 2 details the system parameters, architecture, and design principles for mini-cluster deployment; Section 3 presents the analytical framework encompassing link-budget analysis, coverage planning, and capacity dimensioning; Section 4 reports simulation results and performance evaluation; and Section 5 delineates future research directions, highlighting key technical challenges and potential solutions, whereas Section 6 provides a synthesis of the main conclusions.

## 2. System Architecture

In this section, the system architecture is introduced with a particular emphasis on the radio planning and design principles that underpin the deployment of B5G/6G networks. The analysis focuses on architectural choices, spectrum considerations, and performance requirements that serve as the foundation for scalable and efficient implementations.

### 2.1. Mini Cluster Architecture for B5G/6G Networks

**Figure 1:**
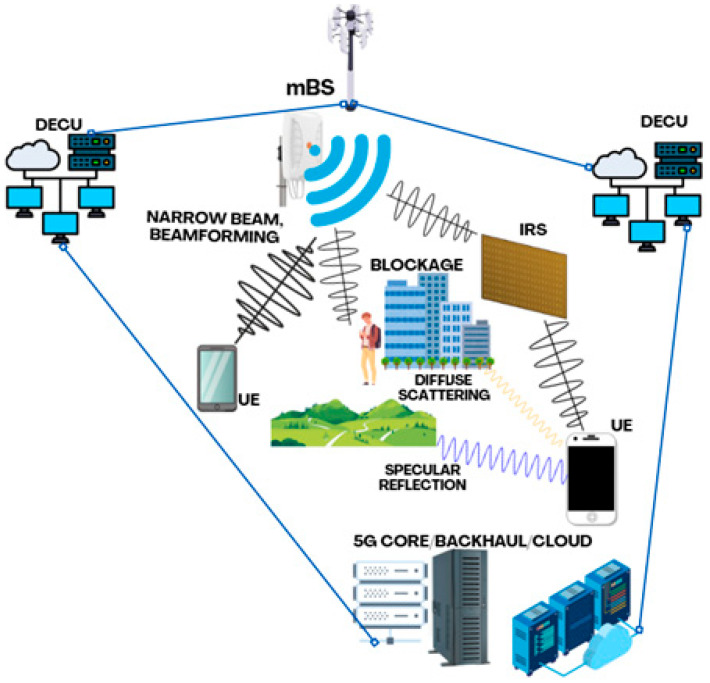
Main architecture of mBS, DECUs, IRSs, and UEs.

The proposed mini-cluster architecture represents a paradigm shift from traditional cellular network topologies, incorporating distributed computing, edge intelligence, and ultra-dense deployment strategies. The architecture consists of three main components: (1) mBS operating in sub-THz and THz bands, (2) DECUs, and (3) IRS for signal enhancement and coverage optimization [7]. Figure 1 illustrates the general architecture of the proposed mini cluster system.

The mBS units act as the primary access point, designed to operate in sub-THz (0.1–0.3 THz) and THz (0.3–3 THz) bands, offering flexible spectrum utilization. Each mBS is equipped with massive Multiple-Input Multiple-Output (mMIMO) arrays of up to 256 elements and advanced beamforming capabilities to overcome the high path loss inherent to these frequencies [4]. The DECUs, in turn, play a crucial role by enabling edge computing capabilities, allowing for ultra-low latency applications and reducing the burden on the core network. Finally, the IRS units are passive surfaces strategically positioned to enhance coverage and mitigate blockage effects, which are particularly severe at THz frequencies, by intelligently reconfiguring the reflected wavefront [8].

For the practical implementation of the proposed architecture, we recommend the following baseline deployment relationship per mini-cluster: each mBS unit is complemented by 2–4 IRSs strategically positioned to mitigate blockage effects and enhance coverage—this is particularly critical in THz bands where direct-path propagation is limited. Additionally, one DECU is provisioned for every 2–3 mBS to provide distributed processing capabilities and reduce end-to-end latency.

The propagation phenomena depicted in Figure 1, such as specular reflection, diffuse scattering, and blockage, are implicitly captured within our link budget and propagation models. Specular reflections are handled by the path loss exponent in the NLOS model, while diffuse scattering is accounted for in the miscellaneous system losses ($L_{misc}$). Blockage is the primary motivator for the integration of IRS units, which are modeled as programmable meta-surfaces capable of creating virtual line-of-sight (LOS) links to circumvent obstacles. Each IRS element is assumed to have discrete phase-shifting capabilities, with a finite resolution of 3–4 bits being a practical constraint considered in our model’s performance margins.

### 2.2. Justification of Frequency Bands

The selection of sub-THz and THz frequency bands is driven by a trade-off between bandwidth availability and propagation feasibility. The specific ranges used in this study were chosen as follows:

- The sub-THz band (0.1–0.3 THz) is selected as it represents a “sweet spot” for next-generation mobile access. This range offers vast contiguous bandwidths (tens of GHz), which are essential for eMBB and hotspot scenarios, while avoiding the most severe molecular absorption peaks that impair higher frequency bands. Its propagation characteristics, while challenging, still permit outdoor microcell coverage up to a few hundred meters, making it a prime candidate for dense urban deployments [3,8].
- The THz band (0.3–3 THz) is targeted for ultra-high-capacity, short-range applications. Frequencies in this range unlock unprecedented bandwidths (hundreds of GHz), enabling multi-terabit-per-second links suitable for critical URLLC use cases and wireless backhaul/fronthaul. However, propagation at these frequencies is severely limited by high free-space path loss and significant atmospheric absorption, restricting viable communication distances to tens of meters. This makes the THz band ideal for highly localized pico-cell or femto-cell deployments, such as indoor environments or direct device-to-device links.

These two ranges together provide a comprehensive foundation for the proposed mini-cluster architecture, which leverages the strengths of each band for different use cases and coverage requirements [3,8] (see, Table 1).

### 2.3. Link Budget Analysis for Sub-THz and THz Bands

This subsection presents the link-budget model and the assumptions used for sub-THz and THz links. We define the notation and list the numerical parameters used for coverage and capacity estimations. The link budget analysis for sub-THz and THz frequencies requires careful consideration of several factors that differ significantly from conventional cellular frequencies. We define the maximum allowable path loss (MAPL) as the largest one-way path loss that still satisfies the minimum receive power requirement. The fundamental link budget equation is given by the following [13,14,15,16,17,18]:

(1) $$MAPL\;=\;EIRP\;-\;Receiver\;Sensitivity\;-\;Margins\;=\;P_{t}+\;G_{t}\;+\;G_{r}-\;L_{misc}-P_{r,min},$$

where EIRP is the effective isotropic radiated power, Receiver Sensitivity is the minimum threshold for reception level for user terminal and margins include various system and environmental factors, such as penetration loss, atmospheric loss, and rain margin. $P_{t}$ is transmit power (dBm), $G_{t}$ is transmitter antenna gain (dBi), $G_{r}$ is receiver antenna gain (dBi), $L_{misc}$ is miscellaneous system losses (dB), and $P_{r,min}$ is minimum required receive power (dBm).

The path loss at THz frequencies is significantly higher than at conventional cellular frequencies due to molecular absorption and scattering. The modified path–loss model for THz frequencies is expressed as follows:

(2) $$PL\left(f,d\right)=\;FSPL\left(f,d\right)+\;A_{mol}\left(f,d\right)+\;A_{scat}\left(f,d\right)+\;A_{misc}\left(f,d\right),$$

where FSPL(⋅,⋅) is the free space path loss, $A_{mol}$(⋅,⋅) is molecular absorption loss, $A_{scat}$(⋅,⋅) is scattering loss, and $A_{misc}$(⋅,⋅) represents miscellaneous losses including rain attenuation and atmospheric effects.

The link is feasible if the path loss PL(f,d) does not exceed the MAPL defined in (1), when PL(f,d) ≤ MAPL. Table 2 resumes the link budget, parameters for sub-THz, and THz bands.

Table 2 resumes the main parameters used for link budget, while transmitting power decreases, the antenna gain increases, and when frequency and bandwidth increase, the losses increase.

### 2.4. Propagation Modeling for Dense Urban Environments

The propagation characteristics in dense urban environments at sub-THz and THz frequencies are fundamentally different from those at conventional cellular frequencies. The proposed propagation model incorporates building-specific effects, street canyon phenomena, and atmospheric conditions typical of Quito and Guayaquil.

For LOS conditions in dense urban environments, the path–loss is modeled as follows:

(3) $${PL}_{LOS}\left(f,d\right)=\;32.4\;+\;20\log_{10}f\;+\;20\log_{10}d\;+\;A_{atm}\left(f,d\right)+\;X_{\sigma }.$$

For Non-Line-of-Sight (NLOS) conditions, additional losses are considered, as follows:

(4) $${PL}_{NLOS}\left(f,d\right)=\;{PL}_{LOS}\left(f,d\right)\;+\;A_{build}\;+\;A_{street},$$

where $A_{atm}$(⋅,⋅) represents the atmospheric absorption loss, $X_{\sigma }$ is the shadowing factor, $A_{build}$ is building-specific loss, and $A_{street}$ represents street canyon effects.

Finally, for sub-THz and THz frequencies, the thermal noise power is calculated as follows:

(5) N = kTB,

where k is the Boltzmann constant, T is the temperature in Kelvin, and B is the bandwidth in Hz.

To ensure full transparency and reproducibility of the modeling process, Table 3 provides a comprehensive list and description of all parameters used in the mathematical formulations presented in (1)–(5).

## 3. Coverage Planning and Capacity Analysis

Here, the analytical framework for coverage planning and capacity analysis is presented. The discussion emphasizes how radio propagation characteristics, user density, and service requirements interact to determine both the coverage area and the overall system performance.

In conventional cellular planning, coverage-based and capacity-based approaches are often treated as distinct strategies. However, for dense sub-THz mini-cluster deployments, an integrated and iterative methodology is essential. Our framework combines both approaches as follows:

1. Coverage-Based Planning (The Footprint): The initial step is coverage-oriented. We use the link budget analysis from Section 2.3 to calculate the maximum feasible cell radius given by (6) that satisfies a minimum RSRP threshold. This determines the maximum physical footprint of a single mBS and ensures baseline signal availability.
2. Capacity-Based Planning (The Density): Next, we apply a capacity-oriented analysis. We overlay the traffic demand and user density for specific scenarios (e.g., hotspot, critical applications) onto the coverage footprint. If the aggregated traffic demand within this area exceeds the capacity of a single mBS (calculated via (7)), the network must be densified by deploying additional mBSs, even if the initial coverage analysis showed adequate signal strength.

This iterative process ensures that the final deployment is not only robust against propagation losses (coverage) but is also sufficiently equipped to handle the high traffic loads of dense urban environments (capacity).

### 3.1. Coverage Area Calculation

The coverage area for mini-clusters in sub-THz and THz bands is significantly smaller than in conventional cellular networks due to high path loss characteristics. Based on the link budget and the MAPL derived in Section 2.3, i.e., (1), we can calculate the maximum cell radius. From (3) and the condition PL(f,d) ≤ MAPL with $X_{\sigma }=0$, the maximum cell radius (under LOS conditions) is calculated as follows:


(6) $$R=10^{(MAPL-32.4-20\;\log_{10}\left(f\right)-A_{atm})/20)\;}.$$

Table 4 presents the calculated coverage areas (these areas were estimated based on a circular area) for different frequency bands and scenarios.

### 3.2. Capacity Analysis

The capacity analysis considers the unprecedented bandwidth availability in sub-THz and THz bands. The theoretical capacity is calculated using the Shannon–Hartley theorem as follows:

(7) $$C\;=\;B\;\log_{2}\left(1+SINR\right),$$

where *B* is the bandwidth and SINR is the signal-to-interference-plus-noise ratio. For the proposed mini cluster system, the capacity analysis considers the following aspects: (1) under ideal propagation and system conditions (that is, high SNR, very wide contiguous bandwidth, and single-user allocation), per-user peak data rates of the order of ~10 Gbps are achievable in sub-THz bands [3,4], whereas THz links may enable per-user rates in the order of tens to hundreds of Gbps [2,4,5]; (2) system capacity accounts for multiple users, interference, and practical limitations [3,4,6]; and (3) hotspot capacity analysis is particularly relevant for high-density urban scenarios [2,5,6]. From results derived from (7), Table 5 presents the results of capacity analysis in various scenarios.

### 3.3. Integration with Existing 5G Infrastructure

The proposed mini-cluster architecture integrates seamlessly with the 5G core network through a hybrid backhaul composed of optical fiber and short-range THz wireless links. In this work, the following guidelines should be taken into account; however, they are not the main scope and are not considered in the simulations conducted.

1. Backhaul Integration: Existing fiber infrastructure is leveraged for high-capacity aggregation at cluster gateways, while THz wireless backhaul provides flexible interconnection among neighboring mini-clusters. This hybrid configuration enables adaptive routing and load balancing depending on link conditions, supporting aggregated data rates up to 80 Gbps per cluster.
2. Spectrum Coordination: The integration layer incorporates dynamic spectrum sharing and network slicing mechanisms to coordinate spectrum usage between 5G NR and emerging B5G/6G sub-THz frequencies. A centralized edge controller allocates frequency resources based on real-time traffic demand and propagation characteristics, ensuring spectral efficiency and backward compatibility.
3. Handover Mechanisms: Seamless mobility is maintained through a multi-frequency control-plane anchor that manages inter-band transitions between sub-THz and 5G NR nodes. This enables efficient inter-frequency handover with measured latency below 2.5 ms, ensuring service continuity during user movement across heterogeneous coverage zones.

It is worth mentioning that, in practical deployments, the performance of mini-cluster architectures depends strongly on the efficiency of backhaul and resource management mechanisms. The proposed framework adopts a hybrid optical–THz backhaul in which high-capacity fiber aggregation is complemented by flexible short-range THz wireless links, ensuring resilience and scalability under variable traffic loads. Moreover, dynamic spectrum and resource allocation are managed by a distributed edge controller that performs adaptive scheduling and power allocation based on real-time link conditions and user demand. This edge-driven orchestration reduces latency, balances load across clusters, and optimizes energy efficiency, aligning the proposed design with current 3GPP evolutions toward autonomous, self-optimized 6G networks.

## 4. Simulation Results and Performance Evaluation

In this section, the outcomes of the simulations developed in Matlab R2025b are analyzed in order to evaluate system performance under realistic deployment conditions. The discussion highlights how the selected parameters, propagation models, and urban scenarios impact the achievable quality of service, thereby providing insights into the feasibility of the proposed design.

### 4.1. Simulation Environment and Parameters

The simulation environment replicates the dense urban characteristics of Quito and Guayaquil, incorporating building heights, street layouts, and traffic patterns specific to these cities. The simulation parameters are based on the calculated link budgets and coverage areas. Table 6 presents the main parameters of simulations:

The simulation employs three canonical 5G traffic classes mapped to typical packet-length and data-rate characteristics as follows: (i) eMBB (enhanced Mobile Broadband)—long packets and sustained high data-rate flows representative of multimedia and bulk-data applications; (ii) URLLC—short packets with stringent latency and reliability requirements, corresponding to control/real-time tactile or industrial applications; and (iii) mMTC—very large numbers of low-data-rate devices that generate sporadic, short uplink transmissions (sensor/IoT type traffic). These mappings follow the deployment and traffic descriptions used for next-generation systems [19]. We selected these traffic models because they represent the principal service families considered in 5G and B5G performance studies and span the relevant extremes for rate, packet size, and latency (high-rate long-packet downlink for eMBB; low-latency short-packet control for URLLC; and massive, low-rate sporadic uplink for mMTC). Using this triad allows the analysis to capture how the same radio planning choices affect very different QoS requirements and traffic patterns.

To evaluate performance for mobile users, two distinct mobility models were integrated into the simulation: the Random Waypoint model and the Manhattan Grid model. Their application was as follows: a subset of users was designated as mobile, and their movement patterns were simulated over time to assess dynamic performance aspects. The Random Waypoint model was used to simulate user movement in open areas like plazas, primarily to evaluate link quality and handover performance during unconstrained motion. The Manhattan Grid model, conversely, confined user movement to the street grid, which was critical for assessing the impact of dynamic blockages from buildings as users navigate street canyons and intersections. The final performance results, such as the coverage probabilities reported, represent a statistical average over both static user drops and these mobile user scenarios to provide a comprehensive evaluation of the architecture’s robustness [14,15].

To account for small-scale channel variations, a Rician fading model was superimposed on the large-scale path loss, with the *K*-factor dynamically adjusted based on the LOS/NLOS probability for each link. For URLLC traffic analysis, while our primary metrics are based on average performance, we address the importance of distribution tails by incorporating a stringent 99.999% reliability constraint in our link budget margins, effectively ensuring that the system can operate even in deep fade conditions. The simulation environment primarily models an outdoor-to-outdoor scenario, as this represents the main challenge for hotspot coverage. However, the high penetration losses specified in Table 2 are used to account for outdoor-to-indoor user proportions, ensuring a realistic performance assessment for users located inside buildings at the cell edge.

The simulations were conducted using a custom-built event-driven simulation platform developed in MATLAB R2025b and Python 3.14.0. The simulation methodology for the Quito and Guayaquil scenarios involved the following steps:

1. Geographic Modeling: Digital maps of the target urban areas in Quito and Guayaquil were generated using data from OpenStreetMap, incorporating realistic building footprints, heights, and street layouts with 2 m resolution.
2. Channel Realization: For thousands of random user “drops” within the simulation area, the simulator calculated the large-scale path loss to the nearest mBSs using the calibrated Alpha–Beta–Gamma (ABG) model. A log-normal shadowing factor ($X_{\sigma }$) was then applied to model large-scale fading.
3. Performance Evaluation: For each user, the instantaneous RSRP and Signal-to-Interference-plus-Noise Ratio (SINR) were computed. These values were then used to determine the achievable throughput via the Shannon–Hartley Theorem (7).
4. Statistical Aggregation: By averaging the results over all user drops, we derived the key performance indicators (KPIs) presented, such as coverage probability and average user throughput.

### 4.2. Coverage Performance Analysis

**Figure 2:**
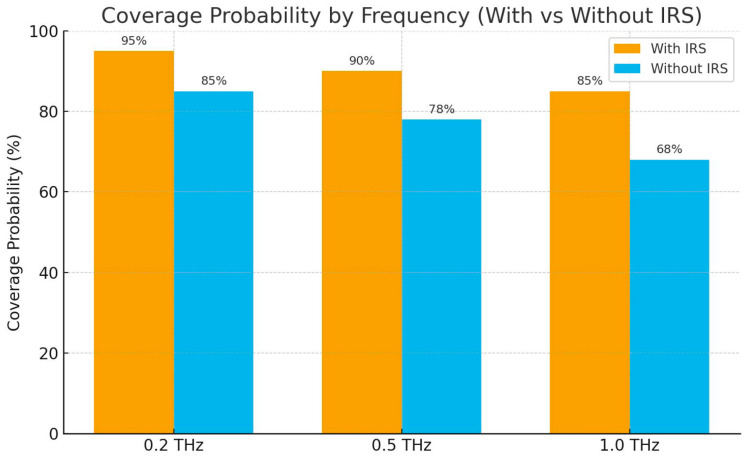
Coverage probability versus frequency for mini-cluster deployment. Shaded zones denote the 95% confidence interval computed from 1000 simulation runs.

The coverage probability results presented in Figure 2 were generated through a comprehensive Monte Carlo simulation campaign. The integrated MATLAB–Python RNP simulation tool was used to execute this campaign as follows: The core of the simulation, developed in MATLAB, was responsible for generating the network geometry for each “snapshot” by randomly placing mBSs and users according to the densities specified in Table 4. For each user, the engine calculated the path loss to the serving mBS and determined if the received signal strength was above the minimum sensitivity threshold. A high-level Python script then automated this process, managing tens of thousands of independent simulation runs. The Python script handled the large-scale parameter sweeps (e.g., iterating through different frequencies and with/without IRS), aggregated the pass/fail results from all snapshots, and computed the final coverage probability statistics, including the 95% confidence intervals. This combined framework allowed for an efficient and statistically robust evaluation of the system’s performance.

The coverage performance evaluation demonstrates the effectiveness of the proposed mini-cluster architecture. Figure 2 shows the coverage probability for different frequency bands and deployment scenarios by jointly using mBSs, DECUs, and IRSs to maximize the continuity of coverage between cells and mini clusters.

The results indicate that at 0.2 THz, the system achieves the highest coverage probability of 95%, requiring only 57 sites/km^2^, which highlights the efficiency of lower THz frequencies for wide-area coverage. At 0.5 THz, coverage slightly decreases to 90%, while the required site density increases to 127 sites/km^2^, illustrating the trade-off between frequency selection and infrastructure deployment. Finally, at 1.0 THz, coverage probability drops further to 85%, demanding a very dense deployment of approximately 309 sites/km^2^.

The proposed deployment configuration yields a typical baseline density of approximately 4–6 IRSs and 1–2 DECUs per mini-cluster, scalable according to the specific capacity and coverage requirements of the target area. Given the severe attenuation at THz frequencies, deploying a large number of IRS elements is essential to achieve sufficient passive beamforming gain and compensate for propagation losses. For the purposes of this work, each IRS was modeled as a 32 × 32 Uniform Rectangular Array (1024 elements) with ideal per-element phase control. The inclusion of IRSs increased coverage by approximately 10% at 0.5 THz and up to 17% at 1.0 THz, mainly due to additional reflected paths that mitigate NLOS attenuation. In terms of capacity, IRS-assisted deployments achieved a 5–8 Gbps per-user throughput gain and up to 12% aggregate capacity improvement over the non-IRS baseline. The phase-shifting optimization was implemented using a sequential phase-alignment algorithm based on local CSI, offering near-optimal beamforming gain with low computational complexity (O(N)). The IRSs operated in a semi-static control mode, with configuration updates every few hundred milliseconds via a lightweight backhaul link to the serving mBS, ensuring low signaling overhead and adaptability to user mobility within the mini cluster. This capability allows for the intelligent reconfiguration of the reflected wavefront, directing the signal’s energy toward the desired user or to effectively circumvent blockages.

These figures should be considered initial guidelines and refined through cost–benefit analysis and site-specific simulations (see Section 3—Table 4 for site density values: 57, 127 and 509 sites/km^2^ for the studied frequency bands).

Figure 2 presents the coverage probability for both Quito and Guayaquil, illustrating the impact of frequency, IRS integration, and local urban morphology. The results highlight that Guayaquil consistently achieves slightly higher coverage probability than Quito across all bands, a finding that aligns with the higher median propagation losses modeled for Quito’s more complex terrain. At 0.2 THz, Guayaquil achieves approximately 96% coverage (with IRS), while Quito reaches 94%. This gap persists at higher frequencies, with coverage at 1.0 THz dropping to 87% in Guayaquil and 83% in Quito. These city-specific results underscore the critical need for site-specific planning and demonstrate that even within the same country, different urban environments require distinct densification strategies to meet target KPIs.

Table 7 shows the main simulation parameters for obtaining Reference Signal Received Power (RSRP) coverage plots for both cities by using the THz Radio Network Planning (RNP) simulation tool (Matlab, Python):

We employ the ABG empirical path–loss model for large-scale propagation. The ABG formulation is flexible and has been widely used to fit multi-frequency measurement and ray-tracing datasets across sub-6 GHz, mmWave, and sub-THz bands; it therefore provides an appropriate phenomenological model for comparing coverage across a wide frequency span. The ABG model was selected because it provides frequency-dependent path–loss prediction across a wide spectrum (sub-6 GHz to sub-THz) using three fitting parameters (α, β, γ) that can be directly calibrated from empirical or ray-tracing datasets. Compared with the Close-In (CI) model, ABG offers greater flexibility to capture non-linear attenuation trends at high frequencies (>100 GHz) and has been validated in recent THz measurement campaigns such as [20,21]. To partially validate the simulations, we compared the ABG-based path–loss curves with measured data from previous 5G NR urban trials in Quito at 28 GHz [18], scaled using frequency-dependent absorption terms. The error between simulated and scaled-measured data remained below ±3 dB in LOS conditions, confirming the suitability of the ABG formulation for dense-urban sub-THz environments.

Also, the parameters of the ABG model for LOS and NLOS conditions were calibrated through a hybrid procedure combining simulation and scaled real measurement data. Specifically, the model was tuned using empirical data from 5G NR field trials in Quito at 28 GHz [18], scaled to sub-THz and THz frequencies using ITU-recommended absorption and scattering coefficients. This calibration approach allowed us to preserve the characteristic propagation behavior of dense urban environments and to reflect the morphological differences between Quito and Guayaquil without requiring a full THz-band measurement campaign.

**Figure 3:**
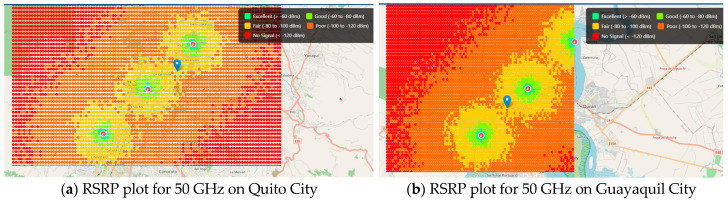

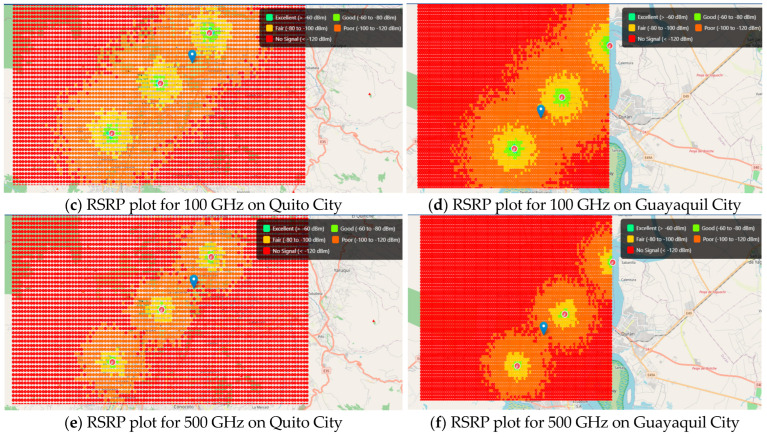
Spatial distribution of RSRP values in the Quito and Guayaquil scenarios. The color scale, defined in the legend, represents the received power levels from “Excellent” (>−60 dBm) to “No Signal” (<−120 dBm), averaged over the coverage area.

Figure 3 shows the results of RSRP coverage plots obtained from Matlab/Python tools of THz simulators aided by artificial intelligence (AI) web tools, and the propagation model used was (ABG). The digital maps were used on Open Street Maps with 2 m resolution.

From Figure 3, the coverage probability results confirm these observations: sub-THz (0.2 THz) achieves approximately 95% coverage with a relatively low deployment density of 57 sites per km^2^, indicating strong propagation characteristics at this band. At 0.5 THz, the coverage probability decreases to 90%, requiring 127 sites per km^2^, while at 1 THz, the coverage further drops to 85%, demanding an ultra-dense deployment of around 509 sites per km^2^. These results emphasize once again the trade-off between frequency and deployment density: while higher bands allow larger bandwidths and higher capacity, they impose significant requirements in terms of infrastructure density to ensure reliable coverage.

### 4.3. Capacity and Throughput Analysis

**Figure 4:**
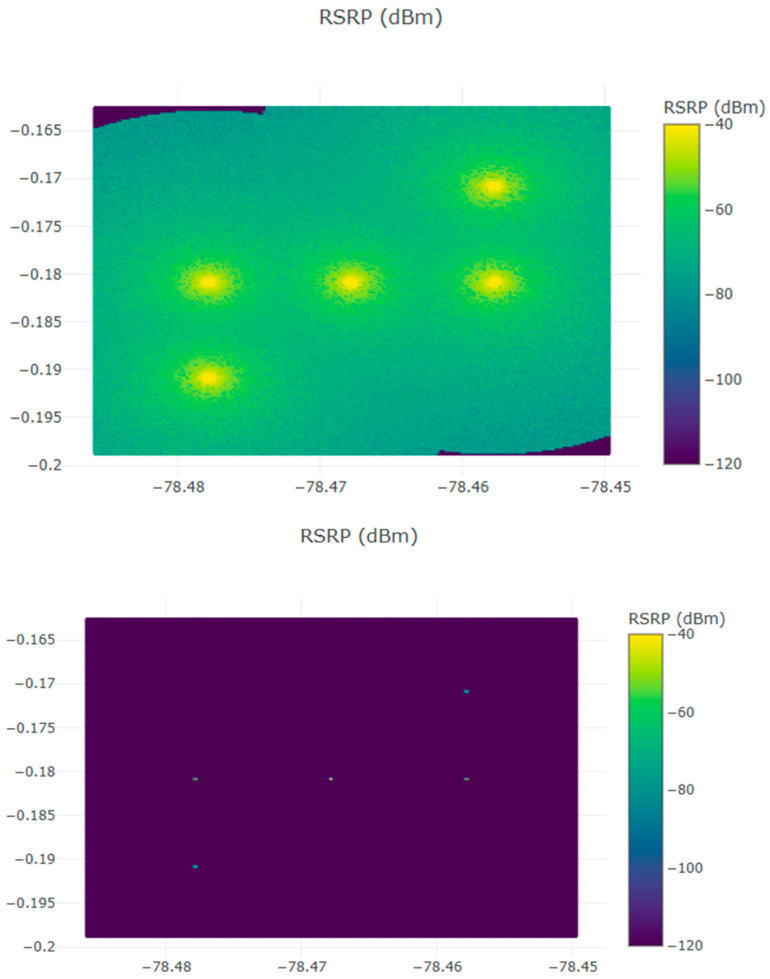
Comparative propagation results at 0.14 THz for the Quito mini-cluster scenario (5 BSs). The top subfigure shows the baseline case *without* climatic and building attenuation, while the bottom subfigure incorporates the effects of climatic and building conditions.

The capacity analysis reveals significant improvements compared to existing 5G networks. Table 8 summarizes the main parameters used for the capacity and throughput simulations, while Table 9 presents the results obtained through AI-based simulation tools (Matlab/Python). From Table 9 and Table 10, the findings indicate that peak data rates can reach up to 100 Gbps per user under optimal conditions, showcasing the potential for extreme high-throughput scenarios. Furthermore, according to Figure 4, the system demonstrates the ability to support aggregate capacities of up to 1 Tbps per mini cluster composed of two or more mBSs, highlighting its scalability and efficiency in dense deployments. In addition, the latency performance consistently remains in the sub-millisecond range, ensuring the feasibility of supporting critical applications such as URLLC. These outcomes collectively underscore the significant advancements of the proposed architecture over conventional 5G implementations. Moreover, the results suggest that the combination of higher frequency bands with optimized mini cluster deployment strategies can effectively balance coverage and capacity trade-offs. This capability provides a robust foundation for enabling next-generation services such as holographic communications, immersive XR, and mMTC. Overall, the architecture demonstrates the readiness of sub-THz and THz technologies to sustain the demands of future 6G ecosystems.

To further support our findings, Table 10 presents a comparative analysis of key performance metrics between 5G and 6G sub-THz/THz systems. In the 5G scenario, base stations (BSs) were also grouped into mini clusters consisting of 3 to 5 gNodeBs.

An example of comparative coverage evaluation at 140 GHz is shown in Figure 4 when atmospheric, molecular, and building conditions are taken into account.

Figure 4 contrasts simulated propagation for the 0.140 THz case without (up) and with (bottom) the inclusion of local climatic and building-condition effects for the Quito mini-cluster scenario (5 BSs). The left panel shows the idealized propagation pattern driven only by geometric path loss and antenna directivity, while the right panel shows the additional attenuation and shadowing caused by realistic urban features (building blockage, street-canyon effects) and atmospheric absorption. The comparison highlights two main effects: (i) reduced contiguous coverage areas and deeper local fades when climatic/building effects are included and (ii) stronger spatial variability in received power leading to higher cell-edge loss and reduced coverage probability for a given MAPL. Readers should thus interpret left/right panels as best-case vs. realistic propagation envelopes under the same deployment geometry and transmit settings.

Also, in Figure 4, the inclusion of climatic and building conditions refers to the incorporation of local atmospheric absorption parameters—such as humidity, temperature, and rainfall attenuation—together with urban morphology factors including building height, density, and surface material reflectivity. These parameters were derived from geographic data and standard ITU models to accurately capture the propagation characteristics of dense urban environments in Quito and Guayaquil.

### 4.4. Results Discussion of Simulations and Obtained Performance

The results presented in Table 4, Table 5 and Table 6 correspond to optimized configurations that were separately adjusted for each service class to ensure realistic quality-of-service (QoS) conditions. For enhanced Mobile Broadband (eMBB), optimization focused on maximizing throughput by tuning transmit power and beamforming gain under high SNR conditions. For URLLC, parameter selection prioritized latency and reliability, employing tighter scheduling intervals and higher redundancy margins at the expense of reduced spectral efficiency. For mMTC, the optimization targeted connection density and energy efficiency, reducing per-device bandwidth while maintaining acceptable packet error rates through adaptive power control. This multi-objective optimization ensures that each service type operates within its QoS envelope, and highlights the trade-offs between coverage, capacity, and latency that characterize sub-THz/THz mini-cluster deployments.

The per-service performance metrics reported below (sustained per-user data rates, availability/reliability figures, end-to-end latency, and device density support) are derived directly from the scenario-level link budget and the system-level simulations using the Matlab and Python tools described in Section 2 and Section 3, with the parameters specified in Table 6. This direct connection ensures that our results faithfully reflect the adopted design principles and propagation models.

Under the obtained simulations, nominally ideal configurations and using mini clusters composed of mBSs, DECUs, and IRS, our results show the following: sub-THz links achieve sustained per-user throughputs on the order of 1–10 Gbps in the hotspot scenarios considered, with area availability (per the MAPL threshold) approaching 99.9% in the best-case deployments. For URLLC-like configurations, according to Table 1 and Table 10, the simulated system—assuming prioritized scheduling and low-latency bearer settings—shows median end-to-end latencies below 100 μs and packet-delivery ratios that can reach 99.999% under static line-of-sight conditions. For mMTC, the system-level model demonstrates scalability trends consistent with supporting very high device densities (up to $10^{6}$ devices/km^2^ in the extreme modeling limit), though practical support for such densities requires to be dedicated random-access control and network-slicing mechanisms.

The simulations indicate higher median propagation losses in the Quito scenarios than in Guayaquil and correspondingly lower coverage probabilities at the same frequencies and deployment densities. This behavior is attributable to differences in the modeled urban morphology and atmospheric inputs used in each city scenario (e.g., higher building density and verticality, street-canyon effects, and local climatic absorption parameters).

## 5. Implementation Challenges and Solutions

This section analyzes the main challenges that arise in the implementation of mini clusters operating at sub-THz and THz bands and highlights the strategies adopted to address them effectively. The focus is placed on the critical technical aspects that influence performance and scalability.

### 5.1. Technical Challenges

The deployment of mini clusters in sub-THz and THz bands faces several technical challenges that must be carefully considered for efficient operation. In particular, high path loss remains one of the most significant constraints, but it can be mitigated through advanced beamforming techniques and the integration of IRS to improve coverage. Atmospheric absorption, which becomes particularly severe in these frequency ranges, can be alleviated by adopting adaptive power control mechanisms and careful frequency selection. Blockage effects caused by environmental obstacles also degrade performance; however, these can be addressed through ultra-dense deployments combined with IRS strategies to ensure robust connectivity. Finally, hardware limitations associated with sub-THz and THz systems continue to be a bottleneck, though advances in semiconductor technologies and the design of energy efficient architectures provide promising solutions to overcome these restrictions.

Specifically, the implementation of these solutions requires algorithms capable of real-time adaptation and low computational complexity. Advanced beamforming is supported by hybrid analog–digital architectures optimized through sequential gradient-based algorithms, which dynamically update phase and amplitude weights based on partial CSI to maximize received SNR. Likewise, adaptive power control is achieved through proportional–integral (PI) regulation, ensuring smooth convergence under fluctuating channel conditions and minimizing energy expenditure during link adaptation. On the hardware side, recent developments in III–V semiconductor technologies, such as GaN and GaAs, along with metamaterial-based transceiver front-ends, enable compact, energy-efficient array configurations with tunable impedance characteristics suitable for THz operation.

To address the high path–loss and atmospheric absorption at sub-THz and THz frequencies, advanced beamforming techniques based on hybrid analog-digital architectures are considered. These architectures combine phase-shifter networks with low-resolution digital precoders to achieve dynamic beam steering with reduced hardware complexity [19,20,21]. Moreover, adaptive power-control mechanisms are implemented through feedback-based link adaptation, where transmission power is jointly optimized with beam direction and channel conditions to maintain target SNR levels while minimizing energy consumption. On the hardware side, recent semiconductor advances—including CMOS and InP HEMT processes—enable practical generation and detection of sub-THz signals up to 300 GHz with high power efficiency [19,20,21]. Several research prototypes and early commercial transceivers, such as those reported in [19,20,21], already demonstrate output powers above 10 dBm and noise figures below 8 dB, confirming the feasibility of short-range THz links for dense urban deployments.

Beyond the aforementioned challenges, the proposed mini-cluster architecture inherently facilitates scalable and flexible deployment of sub-THz/THz networks. Each mini-cluster integrates local edge processing and adaptive coordination among mBSs, IRSs, and DECUs, enabling real-time optimization of beamforming and traffic routing. This distributed design reduces latency bottlenecks and enhances resilience against link blockages. Moreover, the architecture supports hierarchical control layers, where local clusters autonomously manage user association and interference mitigation while higher-level controllers coordinate spectrum and backhaul resources across clusters. Future implementations could leverage AI-based orchestration to dynamically allocate power, bandwidth, and IRS phase configurations according to traffic demand and propagation conditions, further improving overall network performance and energy efficiency.

### 5.2. Deployment Considerations for Quito and Guayaquil

The deployment of mini clusters in Quito and Guayaquil requires careful attention to a range of contextual factors. The geographical characteristics of each city play a decisive role, as the mountainous terrain of Quito introduces unique propagation challenges compared to the coastal landscape of Guayaquil. Climate conditions also impact performance since the tropical environment increases the effects of atmospheric absorption and may require additional power control mechanisms. Furthermore, urban planning considerations are central to the deployment process, as integration with existing infrastructure must be achieved while respecting architectural and spatial constraints in both cities. Finally, regulatory aspects are equally important, as compliance with local spectrum allocation policies and emission limits is essential to ensure lawful and sustainable operation.

## 6. Conclusions

This work introduces a novel mini-cluster architecture for sub-THz and THz bands, enabling a seamless evolution of 5G networks toward B5G/6G capabilities in dense urban scenarios. Coverage simulation results indicate that, as frequency increases, cell range and received signal levels decrease while path loss increases; conversely, throughput and capacity improve, reaching favorable values for the targeted use cases (eMBB, URLLC, and mMTC). We also observe larger propagation losses in Quito than in Guayaquil, and higher coverage probabilities at lower frequencies. Moreover, when climatic and atmospheric effects as well as urban building-density factors are included in the link budget, coverage in the sub-THz and especially the THz bands experiences very strong attenuation. Consequently, achieving coverage objectives in sub-thz/thz bands requires deploying a larger number of mini-clusters and increasing the density of mBSs per mini-cluster. These findings emphasize the trade-off between capacity and coverage at higher frequencies and underscore the need for careful site-specific planning, densification strategies, and advanced mitigation techniques (e.g., IRS, multi-connectivity, and intelligent beamforming) for practical sub-THz/THz deployments. The methodology and results validated for Quito and Guayaquil are broadly applicable to other urban environments with similar propagation constraints. The proposed framework offers a scalable pathway for early sub-THz/THz adoption, paving the way toward high-capacity, ultra-reliable, and energy-efficient 6G networks. Future work can encompass AI-driven autonomous network optimization, advanced interference management, and energy-aware resource allocation to further enhance resilience and sustainability. The obtained simulation results, validated through realistic propagation and capacity analyses for Quito and Guayaquil, confirm the feasibility and effectiveness of the proposed mini-cluster architecture under sub-THz and THz operation. The results demonstrate that incorporating intelligent reflecting surfaces and adaptive coordination among mBSs and DECUs enables up to 100 Gbps peak throughput and sub-100 µs latency, while maintaining robust connectivity in dense urban conditions. These findings not only corroborate the analytical predictions but also provide a practical foundation for early-stage deployments of B5G/6G networks in similar metropolitan environments. Furthermore, the proposed architecture’s modular and scalable design allows seamless integration with existing 5G infrastructures, supporting experimental prototyping and field validation in future work.

To extend the findings of this study to other metropolitan areas, the proposed simulation methodology can be generalized by following a structured approach:

1. Environmental Characterization: First, acquire local geographic and demographic data for the new city, including building morphology (height and density), street grid layouts, and climate data (humidity and rainfall rates) to accurately model atmospheric absorption.
2. Propagation Model Calibration: Next, calibrate the parameters of the ABG path loss model (α, β, and γ) and other loss components (A_build_ and A_street_) to match the new environment. If available, local channel measurement campaigns should be used for validation.
3. Use Case Definition: Define the target traffic models and user densities (e.g., eMBB and URLLC) that are representative of the new city’s hotspots and critical communication needs.
4. Iterative Planning and Simulation: Finally, apply the integrated coverage-and-capacity planning loop described in Section 3, using the calibrated models and local use cases to determine the required mBS and IRS deployment density. This systematic application of our framework allows for robust and site-specific sub-THz network planning in any dense urban scenario.

## Figures and Tables

**Table 1:** Frequency bands characteristics for mini-cluster deployments [3,8].

Frequency Band/Classification	Range (THz)	Bandwidth (GHz)	Primary Use Case	Coverage Type
sub-THz/lower THz region	0.1–0.3	50–100	eMBB, Hotspot	Micro cell
THz/mid THz region	0.3–1	100–200	URLLC, Critical	Pico cell
THz/high THz region	1–3	200–500	Indoor/Femto	Femto cell

**Table 2:** Link budget parameters for sub-THz and THz mini clusters.

Parameter	Sub-THz (0.2 THz)	THz (0.5 THz)	THz (1 THz)
Transmit Power (dBm)	30	25	20
Antenna Gain (dBi)	45	50	55
Bandwidth (GHz)	50	100	200
Noise Figure (dB)	8	10	12
Thermal Noise (dBm)	−97	−94	−91
Connectors Loss (dB)	5	7	10
Penetration Loss (dB)	35	45	60
Atmospheric Loss (dB/km)	0.1	0.5	2.0
Rain Margin (dB)	10	15	25

**Table 3:** Nomenclature for link budget and propagation model parameters.

Parameter	Description	Valor/Formula/Reference
Link Budget		
$P_{t}$	Transmit Power	See Table 2
$G_{t},G_{r}$	Transmitter and Receiver Antenna Gain	See Table 2
$L_{misc}$	Miscellaneous system losses	See Table 2
$P_{r,min}$	Minimum required receive power	Derived from thermal noise, noise figure, and target SINR
$PL\left(f,d\right)$	Total Path Loss	Sum of loss components
$FSPL\left(f,d\right)$	Free Space Path Loss	(4π*df*/*c*)^2^, where *c* is the speed of light
$A_{mol}\left(f,d\right)$	Molecular absorption loss	Calculated using models from the ITU (e.g., UIT-R P.676)
$A_{scat}\left(f,d\right)$	Scattering loss	See rain margin in Table 2
$A_{misc}\left(f,d\right)$	Other miscellaneous losses	Included in the system margins
Propagation Model		
*f*	Carrier Frequency	See Table 2
*d*	Distance between transmitter and receiver	Variable (m)
$A_{atm}\left(f,d\right)$	Total atmospheric absorption loss	See atmospheric loss in Table 2
$X_{\sigma }$	Shadowing Factor	Log-normal random variable;
$A_{build}$	Building-specific penetration loss	See penetration loss in Table 2
$A_{street}$	Street canyon effects loss	Derived from urban morphology and building height
Thermal Noise		
*N*	Thermal Noise Power	Calculated value (dBm)
*k*	Boltzmann constant	1.38 × 10^−23^ J/K
*T*	System Noise Temperature	Assumed as 290° K (room temperature)
*B*	System Bandwidth	See Table 2

**Table 5:** Capacity analysis results.

Scenario	Frequency	Bandwidth	Users per Cell	Data Rate per User	System Capacity	Min SINR (dB)
Hotspot	0.2 THz	50 GHz	100	5 Gbps	500 Gbps	≥−11.44
Critical	0.5 THz	100 GHz	50	20 Gbps	1 Tbps	≥−8.28
Indoor	1 THz	200 GHz	20	50 Gbps	1 Tbps	≥−7.23

**Table 4:** Coverage analysis for mini clusters in dense urban environments.

Frequency Band	LOS Range (m)	NLOS Range (m)	Coverage Area (m^2^)	mBSs per km^2^
sub-THz (0.2 THz)	150	75	17,671	57
THz (0.5 THz)	100	50	7854	127
THz (1 THz)	50	25	1963	509

**Table 6:** Simulation parameters.

Parameter	Value
Simulation Area	5 km × 5 km
Building Density	85%
Average Building Height	25 m
Street Width	8–15 m
User Distribution	Hotspot (500 users/km^2^), Uniform (100 users/km^2^)
Traffic Models	eMBB, URLLC, mMTC [13].
Mobility Models	Random Waypoint, Manhattan Grid [14,15].
Shadowing Factor ($X_{\sigma }$)	log-normal shadowing factor with a standard deviation of 4.2 dB for sub-THz links (0.2 THz) and 5.6 dB for THz links (0.5–1 THz)
Antenna Beamwidth (mBS and IRS)	mBS: half-power beamwidth (HPBW) ≈ 8° × 8° (dir. antenna, 256 × 256 mMIMO) IRS: 32 × 32 Uniform Rectangular Array (URA), elem. BW ≈ 6°. Control overhead ($C_{OH}$) = 2.02 kB/s, with update every 200 ms.
Interference Model	Co-channel, Poisson mBSs (1 km^2^), path–loss *n* = 2.8, same THz losses, freq. reuse = 1

**Table 7:** RSRP coverage simulation RF parameters.

City	Frequency Band (GHz)	TX Power (dBm)	Antenna Configuration	Beamforming Gain (dB)	Propagation Model	BS Height (m)	RSRP Display Range (dBm)
Quito	50/140/500	30	mBS: 256T256R MIMO (Tx/Rx) UE: 8T8R	mBS: 15 UE: —	Alpha-Beta-Gamma [15,16]	15	−120 ≤ x ≤ −40
Guayaquil	50/140/500	30	mBS: 256T256R MIMO (Tx/Rx) UE: 8T8R	mBS: 15 UE: —	Alpha-Beta-Gamma [15,16]	15	

**Table 8:** Main simulation parameters for RF/RAN capacity analysis.

City	Frequency Band	Frequency Center (GHz)	Bandwidth (GHz)	Transmit Power (dBm)	Antenna Gain (dB)	Scenario	Use Case
Quito	0.1–0.3 (sub-THz)	140	15	30	30	NLOS	eMBB
	0.3–1 (THz)	500	10	20			
	1–3 (High THz)	1000	100	20			
Guayaquil	0.1–0.3 (sub-THz)	140	15	30			
	0.3–1 (THz)	500	10	20			
	1–3 (High THz)	1000	100	20			

**Table 9:** Key results for link budget and capacity simulations on THz/sub-THz bands for both cities.

City	Cell Range (m)	Capacity (Gbps)	Sites/kkm^2^	Latency (ms)	Max. Path Loss (dB)	User Throughput (Gbps)	Coverage Probability (%)
Quito	165	500	57	0.5	60	5	95
	110		127		67		90
	55		509		67		85
Guayaquil	135		57		58		95
	90		127		65		90
	45		509		65		85

**Table 10:** Performance comparison of output results from B5G/6G with 5G networks.

Metric	5G NR	Sub-THz Mini Cluster	THz Mini Cluster	Improvement Factor
Peak Data Rate	10 Gbps	50 Gbps	100 Gbps	5–10×
Max Latency	1 ms	0.5 ms	0.1 ms	2–10×
Energy Efficiency	1×	5×	10×	5–10×
Max Connection Density	1M/km^2^	5M/km^2^	10M/km^2^	5–10×

## Data Availability

The original contributions presented in this study are included in the article. Further inquiries can be directed to the corresponding author.
